# MR-Guided Radiotherapy for Liver Malignancies

**DOI:** 10.3389/fonc.2021.616027

**Published:** 2021-04-01

**Authors:** Luca Boldrini, Stefanie Corradini, Cihan Gani, Lauren Henke, Ali Hosni, Angela Romano, Laura Dawson

**Affiliations:** ^1^ Dipartimento di Diagnostica per Immagini, Radioterapia Oncologica ed Ematologia, Fondazione Policlinico Universitario “A. Gemelli” IRCCS, Roma, Italy; ^2^ Department of Radiation Oncology, University Hospital, LMU Munich, Munich, Germany; ^3^ Department of Radiation Oncology, University Hospital and Medical Faculty, Eberhard Karls University, Tübingen, Germany; ^4^ Department of Radiation Oncology, Washington University in St Louis, St Louis, MO, United States; ^5^ Radiation Medicine Program, Princess Margaret Cancer Centre, Department of Radiation Oncology, University of Toronto, Toronto, ON, Canada

**Keywords:** magnetic resonance guided radiotherapy, stereotactic body radiation therapy, image guided radiation therapy, liver malignancies, liver cancer, online adaptive radiation therapy

## Abstract

MR guided radiotherapy represents one of the most promising recent technological innovations in the field. The possibility to better visualize therapy volumes, coupled with the innovative online adaptive radiotherapy and motion management approaches, paves the way to more efficient treatment delivery and may be translated in better clinical outcomes both in terms of response and reduced toxicity. The aim of this review is to present the existing evidence about MRgRT applications for liver malignancies, discussing the potential clinical advantages and the current pitfalls of this new technology.

## Introduction

The recent introduction of integrated magnetic resonance (MR) linear accelerators (linacs) into clinical practice has opened new perspectives for radiation therapy (RT), offering the advantages of coupling 0.35 or 1.5 T on-board MR scanners firstly with a triplet of ^60^Co heads and later with 6 and 7 MV linacs in stand-alone hybrid units (1–3). MR guided radiotherapy (MRgRT) has been successfully applied to several anatomical sites, exploiting online adaptive planning solutions and innovative motion management, with improved dosimetric performance and early clinical results suggesting improved efficacy and toxicity reduction (4–6). Despite the numerous explored applications, the published clinical evidence is still scarce, and the actual quantification of the advantages of using such an advanced technology is still the object of debate in the radiation oncology community (7). The types of cancers generally considered most suitable for MRgRT are those located in anatomical sites where similar levels of tissue density in computed tomography (CT) imaging do not allow a precise identification of the different therapy volumes, especially if they are movable and particularly close to radiosensitive organs at risk (OAR).

In this framework, liver malignancies appear to be ideal for MRgRT applications for several reasons, especially when considering the growing role that stereotactic body radiation therapy (SBRT) is gaining in the treatment of both primary liver tumors or liver metastases (8–10). MRgRT could indeed be a competitive option to improve tumor control, especially during hypofractionated radiotherapy and for tumors that are poorly visualized on standard radiotherapy CT imaging (*i.e.* liver cancers). Furthermore, the innovative online adaptive solutions have made it possible to dose escalate to ablative doses even for targets close to sensitive OARs (*e.g.* bowel loops, duodenum, stomach) (4, 8, 11–13).

The aim of this article is to describe the state of the art of MRgRT for liver tumors, focusing on the most promising liver cancer clinical indications, the role of the different MRI sequences provided by the hybrid scanners, and the advantages of applying motion management and advanced adaptive approaches using MRgRT.

## Liver MRgRT Clinical Indications

The role of RT in the management of primary and secondary liver tumors has substantially increased over the years. Emerging data suggest local treatment benefit for both hepatocellular carcinoma (HCC) and oligometastatic disease, integrating radiation therapy (RT) in different ways with available systemic and local therapies (14). In both the aforementioned disease conditions, the treatment of choice is surgery with 5-years survival rates of 30–60% for colorectal cancer (CRC) liver metastases and 50% for HCC, with 4-years survival of 74% after liver transplantation (15–17). Other liver-directed treatments, such as radiofrequency ablation (RFA), interstitial brachytherapy (IBT), microwave ablation (MWA), or percutaneous ethanol injection (PEI) are valid treatment options for small tumors when surgery is not possible, *e.g.* due to comorbidities or limited liver reserve (17). Transarterial chemoembolization (TACE), Yttrium-90 (^90^Y) transarterial radioembolization, and drug-eluting bead transarterial chemoembolization (DEB-TACE) are regional, non-curative therapies used to improve survival in selected HCC patients (18, 19). Many patients with liver cancers are not well suited for these local–regional therapies, and many others develop recurrences despite the use of these therapies. Thus there is a potential role for RT to be used to treat these patients who may not be treated with ablative therapies otherwise. Liver radiotherapy has been historically used for palliation, but its therapeutic paradigm is changing, in part due to the application of SBRT which allows a high conformation of the dose to the target with efficacious sparing of the OARs and significant reduction of the risk of radiation-induced liver disease (RILD), which represents an important cause of comorbidity, especially for primary liver cancers (20).

### Primary Liver Lesions

Numerous trials have demonstrated the effectiveness of SBRT in primary liver cancers, but there is still no conclusive scientific evidence to definitely determine the role and benefits of RT in this setting (21, 22). SBRT plays a major role mainly when surgery or other local ablative procedures (*e.g.* RFA) are contraindicated or high risk. Such patients may have early stage tumors with a high chance of sustained local control, *e.g.* HCC early stage by the Barcelona Clinic Liver Cancer (BCLC) classification: solitary lesions ≤5 cm in maximum diameter or multiple nodules (≤3 total) measuring ≤3 cm in maximum diameter, absence of vascular invasion and extra hepatic metastasis (23). SBRT can also be used as a salvage treatment after other local therapies have failed (23). Alternatively, SBRT has an increasing role in intermediate and advanced stage tumors, where avoiding toxicity is important.

The feasibility and effectiveness of SBRT have been demonstrated in comparative studies between SBRT and RFA and between SBRT in combination with TACE *versus* SBRT alone, without negative impact on the toxicity profile (24–26). Particular caution should be used for patients with more impaired liver function, *e.g.* Child Pugh score >8 points, reserving SBRT only as a bridge to transplantation, since a correlation with increased liver toxicity has been reported in these patients subset (27–29).

Encouraging results of SBRT on survival and toxicity have also been reported in patients where TACE and surgery are contraindicated due to the presence of portal vein tumor thrombosis (PVTT) (30, 31). Small series have reported results following the combination of SBRT with Sorafenib, a multikinase inhibitor targeting the Raf/MEK/ERK pathway, and caution is suggested in this subset of patients due to the possible increase of hepatic toxicity for possible post irradiation impairment of normal tissue recovery process secondary to anti VEGF activity (32–34).

Immunotherapy, in particular, therapies targeting PD-L1-PD-1 pathways (*i.e.* checkpoint inhibitors, Atezolizumab) and antibodies targeting vascular endothelial growth factor (VEGF), is taking on an emerging role. The combination of atezolizumab and bevacizumab has been shown to result in better OS and PFS outcomes than Sorafenib in patients with unresectable HCC (35).

Lastly, even if not supported by robust evidence, some published experiences also suggest a potential role for SBRT also in the management of cholangiocarcinoma, especially when combined with systemic therapies (36).

### Liver Secondary Lesions

SBRT plays an important role also in the management of non-resectable oligometastatic liver disease, and several studies have demonstrated the role and effectiveness of SBRT as a non-invasive, well-tolerated, and promising therapeutic approach, especially in the light of the aforementioned technological progress represented by MRgRT.

Hypofractionated regimens have been adopted for some time now, showing promising results on local disease control, but the potential for unnecessary high dose OAR irradiation, linked to increased rates of toxicity, has limited widespread use of SBRT (37–40). The optimization of traditional SBRT delivery technologies (*i.e.* Cone beam CT, CBCT, IGRT protocols, and fiducial based irradiation) has achieved better local control rates for small lesions, reporting local control rates >90% when doses of 46–52 Gy are delivered in three fractions for unresectable colorectal metastases (41, 42). Dose escalation appears therefore to be directly linked to local control and clinical outcomes, and MRgRT may ensure higher degrees of safety and efficacy.

Multidisciplinary assessment is recommended to identify patients who may be eligible for SBRT, based on location, size, and morphology of liver lesions and on patient performance status, liver function, and residual healthy liver volume (42). Careful selection of patients for ablative therapies is required when there is a potential risk of RILD or when patients have comorbidities that contraindicate invasive treatments. SBRT can be used for metastatic lesions that are challenging to be treated with RFA due to their proximity to critical structures (*e.g.* subcapsular, periampullary, perihilar or when adjacent to vascular structures). An advantage was shown in terms of 1-year freedom from local progression (FFLP) when SBRT is compared to MWA when larger lesions are treated (43, 44). Furthermore, recent data encourage the use of RFA and SBRT for the management of multiple liver metastases (45).

### Clinical MRgRT Liver Evidence

Rosenberg et al. (11) and Feldman et al. (46) have focused on the feasibility of MRgRT in the treatment of both primary and secondary hepatic neoplasms. Rosenberg et al. (11) analyzed the outcomes of 26 patients treated with MRgRT SBRT technique in different institutions. Patients with both Child–Pugh A or early B and presenting one to three liver lesions were included. Median PTV was 98.2 cc (13–2,034), with a median delivered dose of 50 Gy in five fractions, and median liver dose of 12.7 Gy (3.2–21.9). The applied gating protocols were deep inspiration breath hold (DIBH) (16 patients) and modified shallow internal target volume or exhale-based setup for treatment (10 patients), depending on the patient’s compliance. At 21 months follow-up, local control rate was 80.4% with grade 3 gastrointestinal toxicity found in two patients (7.7%, with one case of portal hypertension and one of hilar stricture requiring procedures) who had a large treatment volume and had undergone previous liver-directed treatments. The 1-year and 2-years OS were 69 and 60% respectively.

In the cohort of 29 patients treated by Feldman et al. (46), 26 were affected by HCC, two by cholangiocarcinoma, and one presented liver metastases. A total of 31 lesions were treated with a dose ranging from 45 to 50 Gy in five fractions, while the remaining three were treated with doses from 27 to 42 Gy in three fractions. The mean liver dose was 5.56 Gy (1.39–10.43). Motion was managed by treating 21 patients in end-exhale, six in end-inhale, and two in free breathing conditions. One patient was also treated with adaptive technique. Patients were monitored in follow-up from one to 12 months post-treatment, showing either stable or decreased size of all but one treated lesion. The highest observed toxicity was grade G2 with a case of nausea and vomiting and a case of abdominal pain with melena that did not require pharmacological intervention, but only a brief interruption of treatment.

Moreover, Henke et al. (47) reported the potential of the Stereotactic MR-guided adaptive radiation therapy (SMART) approach (48) in a cohort of oligometastatic patients including 11 patients affected by secondary liver lesions and four with HCC. At median follow-up of 15 months only two patients with recurrent locally advanced pancreatic cancer underwent local progression. No grade 3 toxicity has been observed in this cohort of patients, while 6-months local progression free survival rate and 1-year OS were of 89.1 and 75% respectively. Hal et al. (49) recently presented data from a cohort of 10 patients affected by upper abdominal neoplasms (of whom four were affected by secondary liver lesions and two by HCC), treated with 1.5 T MR-linac.

HCC patients received 40–45 Gy in five fractions, while those with metastatic lesions 45–60 Gy in three fractions or 50 Gy in five fractions. A 4DCT and a 4DMRI with IV contrast agent were acquired in the simulation phase.

Motion was managed creating an ITV from the 4DCT simulation. Treatment has been carried out with both adapt-to-position (ATP) and adapt-to-shape (ATS) approaches, and the delivery has been performed with a real-time cine MRI acquired in three perpendicular planes. At 7.2 months follow-up, two patients developed G2 skin toxicity, and no local recurrences or progression of the treated lesions was recorded. The feasibility and patients’ acceptability of MRgRT were investigated in a prospective study that enrolled 43 patients, including eight with liver lesions, who underwent respiratory-gated treatments in DIBH, of which 47% SBRT (50). The treatment was carried out with visual guidance of the live sagittal low T cine-MRI during gated delivery coupled with audio feedback when necessary. Patients compiled an in-house developed patient-reported outcome questionnaire to document their treatment experience and tolerance. Although 65% of patients reported some MR-related complaints (*e.g.* paraesthesia, uncomfortable positioning), MRgRT was overall defined as positive or at least tolerable.

All patients reported high levels of satisfaction related to their active participation in treatment. No acute toxicity ≥G2 was recorded in the entire cohort, except for four patients reporting G2 fatigue.

**Table 1** summarizes some of the clinical studies on the use of MRgRT in the treatment of hepatic malignancies.

**Table 1:** Recent clinical studies on the role of MRgRT in hepatic malignancies.

Reference	year	dose	Patients (n)	Response
Henke et al. (51)	2018	50 Gy in 5 fractions	10 non-liver abdomen lesions 6 MLL 4 HCC	3-months LPFS 95% 6-months LPFS 89.1% 1-year OS 75%
Feldman et al. (46)	2019	45 to 50 Gy/5 fractions	26 HCC 2 cholangiocarcinoma 6 MLL	1 year LC 96.5% 1 year OS 92.8%
Rosenberg et al. (11)	2019	Median dose 50 (30–60) Gy in 5 fractions	6 HCC 20 MLL	1-year OS 69% 2-years OS 60%
Hal et al. (49)	2020	Median dose 45 (25–60) Gy in 3 to 5 fractions	3 Pancreatic cancer 2 HCC 1 pancreatic metastasis 4 MLL	7.2-months LC 100%
Luterstein et al. (52)	2020	Median dose 40 Gy in 5 fractions	17 cholangiocarcinoma	1-year OS 76% 2-year OS 46.1% 1-year LC 85.6% 2-year LC 73.3%.
Boldrini et al. (53)	2021	Median dose of 50 (50–55) Gy in 5 fractions	10 HCC	6,5-months LC 90%
(ClinicalTrials.gov. NCT04242342) (54)	2019– recruiting	50–60 Gy in 5 to 6 fractions	46 Primary or secondary liver tumor(s)	2 years LC Lack of progression according to RECIST criteria

MLL, metastatic liver lesions; OS, overall survival; LC, local control; LPFS, local progression free survival.

## MRI Imaging Characterization

The reliable identification of liver lesions on hybrid MR imaging depends on several issues and has direct consequences in RT treatment planning (*i.e.* planning target volume, PTV, margin definition, and gating solutions). Magnetic resonance scanner field strength, presence of image artefacts (especially respiratory related ones), used sequence, and the administration of contrast agents should be considered among the technical ones. Other clinical and patient’s specific parameters have specific consequences on image quality and reliability for radiotherapy segmentation and planning purposes, such as the kind of disease (primary liver tumors or secondary lesions), the involved hepatic segment or specific anatomical conditions. MR-linacs currently allow the acquisition of a default sequence which is similar to the standard “true-FISP”, both in the 0.35 and 1.5 T clinical solutions. This sequence generally allows tumor identification and easier segmentation of the upper abdominal OAR, representing an advantage when compared to kV CBCTs (55). Favorable experiences regarding the visibility of metastases and primary liver cancer have been reported for both low and high field MR-linac hybrid devices (46, 49). Furthermore, the use of contrast agents or specific sequences enriches the standard positioning image and allows better visualization of the OAR. Liver specific contrast agents such as gadoxetic acid (*i.e.* Gd-EOB-DTPA or Gd-BOPTA) are eliminated through the biliary tract and lead to a bright appearance of the liver, therefore improving the contrast between healthy and tumorous liver tissue and offering a better visualization and characterization of the lesions in late hepato-specific phase (56). Such agents have also been used in the context of clinical online MRgRT; however, caution is warranted with the repeated application of contrast agents within a short time frame, and safety data are still scarce about possible toxicity. When clinically indicated, MR compatible fiducials may also be implanted as reference markers: platinum ones have the most favorable technical and logistic profile (57). Imaging and sequence comparison studies between diagnostic and hybrid MRI are still lacking, and the need to rely on standard diagnostic imaging, especially for target volume segmentation support, is currently still strongly suggested for MRgRT applications.

### Primary Liver Cancers

HCC nodules show great variability in imaging characteristics and radiological aspects due to the varying content of substances. Their semeiotics in T1WI and T2WI is generally not constant, and the acquisition of dynamic contrast is a key factor for diagnosis and tumor characterization, especially to detect vascular invasion (58).

**Figure 1:**
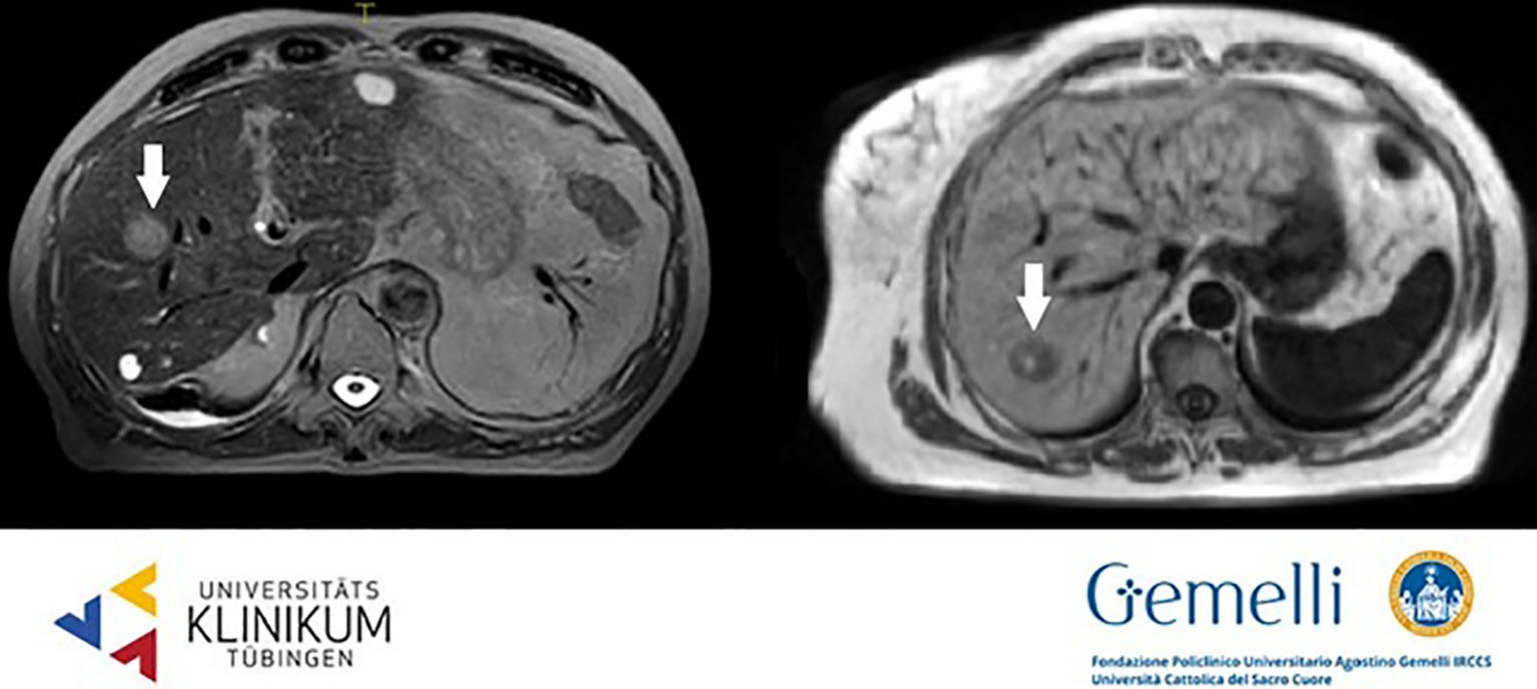
HCC nodules on T2 weighted 1.5 T hybrid imaging (left) and on T1 weighted 0.35 T hybrid imaging (right).

HCC usually shows early arterial phase enhancement and rapid washout in the portal venous (PVP) or delayed phases (DP), while it is generally hypointense in the hepatobiliary phase (HBP) (59). The use of T2WI and DWI may be useful to make differential diagnosis between uncommon hyperintense HCC presentations or focal nodular hyperplasia or other benign conditions. The semeiotics of HCC nodules in standard 0.35 T TRUFI imaging is mixed with prevalence of hyper-isointense aspect. **Figure 1** shows HCC lesions on hybrid MRgRT images for both high and low tesla units.

The radiological aspect of cholangiocarcinoma on MR imaging depends on the anatomical site and on its growth characteristics and may be successfully described using complex magnetic resonance studies including cholangiopancreatography, conventional T1WI, T2WI, DWI, and Dynamic Contrast Enhanced (DCE) sequences. Peripheral mass-forming intrahepatic presentations generally appear isointense or moderately hypointense in T1WI and hyperintense in T2WI, with restricted diffusion in DWI. Contrast enhancement is characteristically late and centripetal and may facilitate the differential diagnosis from other masses (*i.e.* HCC and metastases). Periductal infiltrating lesions are visible on T2WI showing hyperintense dilatation of the upstream ducts, while extrahepatic ductal forms generally appear as masses to be differentiated from pancreatic head adenocarcinomas (60). Cholangiocarcinomas are generally hypointense in TRUFI on 0.35 T hybrid units.

### Secondary Lesions

**Figure 2:**
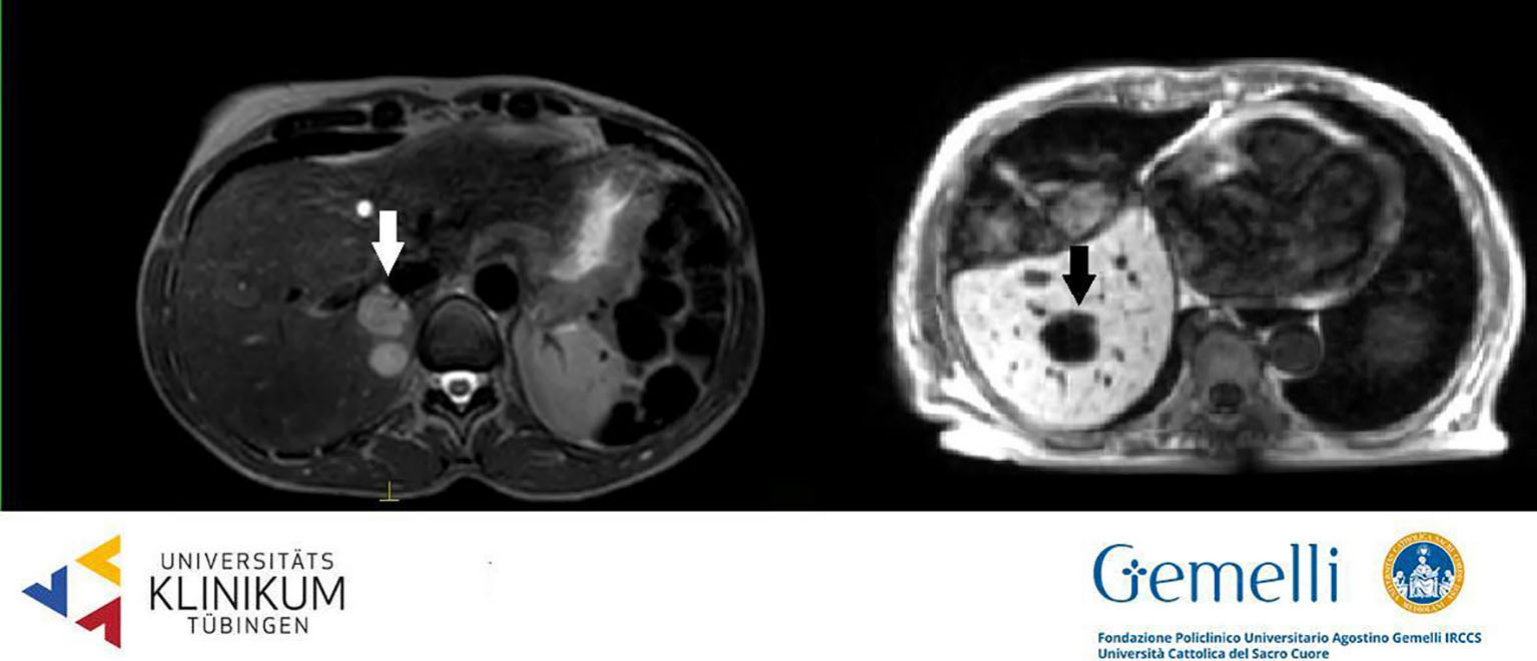
Liver secondary lesions on T2 weighted 1.5 T hybrid imaging (left, hyperintense, from breast cancer) and on T1 weighted 0.35 T hybrid imaging (right, hypointense, from gastric cancer).

Liver metastases are generally hypointense in the HBP, appearing as areas of loss of signal with respect to the enhanced normal liver parenchyma, due to cellular substitution (61). The radiological semiotics of secondary hepatic lesions may suggest the originating disease, thanks to specific image characteristics. Adenocarcinomas metastases appear hypointense in T1WI, slightly hyperintense in T2WI, with restricted diffusion and low apparent diffusion coefficient (ADC) values. The use of contrast agents generally discloses a hypovascularized central core accompanied by a hypervascularized external rim (62, 63). Pronounced hypervascularization in dynamic phases is characteristic also of neuroendocrine tumors (NETs); melanoma, thyroid, and renal cancer more often show a hypervascularized aspect (64–66). On the other hand, colorectal, lung, and breast cancer secondarisms generally appear hypointense compared to the enhancing normal liver parenchyma in PVP. Secondary liver lesions generally appear as hypo-isointense nodules in standard 0.35 T TRUFI positioning image (see **Figure 2**) (63).

**Table 2** summarizes the sequences of more common clinical use for liver target volumes identification.

**Table 2:** Liver lesions in the different MR sequences in current MRgRT clinical use.

Lesion	T1WI (non-CE)	T2WI	TRUFI (0.35 T)
Hepatocarcinoma	Hypointense	Iso-hyperintense	Iso-hyperintense
Cholangiocarcinoma	Hypointense	Iso-hyperintense	Iso-hypointense
Metastases	Hypointense	Hyperintense	Hypointense

CE, contrast enhanced.

## MRI Based RT Volume Segmentation

The standardized and accurate definition of target volumes and OAR has become an even more crucial factor in the MRgRT workflow. For instance a relevant organ at risk delineated erroneously too large might prevent sufficient target volume coverage in the daily adaptive workflow and, *vice versa*, severe toxicity may result if OARs are not delineated at their full extent. For this reason, a panel of radiation oncologists and radiologists with experience in the field of online MR guided radiotherapy of the liver has recently published an atlas for OAR contouring in the upper abdomen (55). Dicom datasets with recommended delineations of upper abdominal OAR structures can be found at www.econtour.org. More specifically, when contouring the liver on MRI it is recommended to exclude the inferior vena cava and include the caudate lobe in order to achieve an appropriate quantification of functional liver tissue. Both structures are challenging to identify on non-contrast enhanced computed tomography simulation scans but can well be visualized on both T1WI and T2WI. Another structure that is sometimes poorly visible on CT scans is the common bile duct. *Post hoc* studies of hepatobiliary toxicities, such as biliary structures or elevated liver function tests after SBRT for centrally located tumors, suggest a dose effect for these toxicities (67). The common bile duct can be clearly seen on T2WI in most cases or on the HBP after the application of liver specific contrast agents. The delineation of this structure might help to prevent these toxicities by considering them during plan adaption and to further improve our knowledge about the dose–volume relationship in this anatomical site (68). Stomach, duodenum, and small bowel loops are the most critical OAR when high doses of radiotherapy are applied in the upper abdomen. In most instances a T2 weighted scan is the optimal sequence for their delineation; however due to motion artifacts caused by peristalsis there is still a need for optimized sequences in adaptive MR-linac workflows and OAR margin definition indications. The administration of a glass of water shortly before the treatment fraction may help in visualizing the stomach and the duodenum (that will appear hyperintense in TRUFI and T2 images), while the use of antiperistaltic agents (*e.g.* butylscopolamine) may reduce the motion related artefacts allowing a more efficient and reliable segmentation process.

## Motion Management for Liver MRgRT

SBRT is characterized by the attempt to minimize PTV and to provide a rapid dose fall off towards the surrounding healthy tissues. Especially in liver SBRT, the main challenges are the proximity of the tumor to many vulnerable OARs such as the healthy liver, duodenum, stomach, bowel, kidneys, or spinal cord and the mobility of both the tumor and the surrounding OARs triggered by breathing-related motion or by changes in the filling status, anatomical arrangement or deformation of gastrointestinal organs (69). Organ motion in the abdominal region is greater than in other sites, with movements in the cranio-caudal direction of up to 4 cm, which is two to three times larger than the movements in the anterio-posterior or lateral directions (69, 70). This is often compensated by an increase in the irradiated internal target volume (ITV concept) (71), which on the other hand can be accompanied by the trade-offs of losing the potential gain of modern radiation techniques in sparing OARs. In liver radiotherapy, the post-interventional liver function can be predictive for patient survival (72). Therefore, adherence to radiation tolerance of normal liver tissue and keeping the associated risk of RILD to a minimum are of utmost importance.

Available motion management strategies to compensate for intrafractional breathing-related organ motion in conventional image-guided liver SBRT can be categorized into: 1) non-gated techniques (with or without mechanical abdominal compression) using the adoption of the ITV or mid-position concept; 2) respiratory-gated techniques, including use of a breath-hold ‘immobilization’ approach; or 3) real-time tumor tracking (73, 74). Due to the relatively poor soft tissue contrast in conventional SBRT using CBCT, frequently the tumor cannot be directly visualized, and implantation of fiducial markers next to the tumor or other surrogates is needed to facilitate image-guidance (75, 76).

In this setting, the application of MRgRT marks the beginning of a new era, as multiple features of this new technology may improve the application of liver SBRT and enable dose escalation strategies, and reduced doses to adjacent normal tissues. Besides the advantages of online treatment plan adaption strategies, which will be highlighted in the next section, the technology enables a direct visualization of the target—even during treatment delivery (4, 77). With currently available MR-linac systems, continuous real-time 2D-cine-MRI is used to assess tumor motion (2, 78). In future, also three-dimensional (3D) MR scans at an adequate resolution and frame rate to monitor fast motion might be available and further improve the applicability of MR guidance for intrafractional motion monitoring (*i.e.* 4D or respiratory correlated MR) (73). To date, the Viewray system also allows automated gating by using repeated fast planar cine-MRI in a sagittal plane with four to eight frames per second (2). This eliminates the need for invasive implantation of fiducial markers as well as the application of ITV in order to account for intrafractional motion (50).

Early experiences show promising results (8, 11, 46). Rosenberg et al. (11) report on a multi-institutional experience of MR-guided SBRT using a 0.35 T MR-linac system. Respiratory-gated SBRT was performed by using a voluntary breath-hold procedure without any external respiratory motion management system. Simulation with real-time sagittal TRUFI cine MRI sequences was used to evaluate tumor motion and to find a reproducible and tolerable breath-hold level. The breath-hold technique requires that the patient inhales to a specified threshold and successively holds the breath at a specific level of inspiration during delivery of every radiation beam. This enables minimization of tumor movement and allows for a reduction of the irradiated liver volume. While the breath-hold technique is usually performed in deep inspiration for thoracic tumors, a shallow breath-hold or expiration breath-hold seems also feasible to mitigate target movement for upper abdominal tumors, like liver tumors. In this setting, the breath-hold technique has proven to be a safe and effective way to reduce tumor motion, resulting in an average intrafractional movement of <1 mm in all directions and an average cranio-caudal interfractional reproducibility of <4 mm (79, 80). Some authors reported on the implementation of an additional visual feedback for the breath-hold procedure using in-room screens or projectors (81, 82). This allows patients to see their live cine MR images including projections of target and gating boundary and, thus actively control their breathing.

During RT delivery, the 0.3 T MR-linac system can automatically gate the beam by using real-time anatomy structure tracking (83). For this purpose, a target structure is defined in the sagittal view of the volumetric MRI, and a surrounding gating boundary contour is created by adding an appropriate tracking margin. Usually, the gating boundary is equal to or less than the PTV margin. The tracking algorithm deforms the anatomical contour on every subsequent live cine MRI frame and compares it to the static boundary contour. If the anatomy of interest moves outside the boundary, the beam is stopped until the tracked anatomy returns into the boundary. The percentage of the target that may be outside the boundary before beam is shut-off can individually be adjusted. The vendor-defined specification for the gating latency of the 0.3 T MR-linac system is <500 ms (2).

The target structure used for tracking is usually the liver tumor itself. Nevertheless, some liver tumors are often poorly visualized, even in MR imaging. Therefore, the application of hepatocyte-specific contrast agents, such as gadoxetate disodium, is reported to significantly improve visualization of liver lesions during simulation and real-time MR-guided SBRT (84). If visualization is still not optimal, tracking can also be performed on surrogate structures, such as the portal vein, liver contour, or other anatomical structures (11). The Unity system is likely to have this capability soon, but at present can only gate the beam manually.

Taken together, MRgRT using a respiratory-gated SBRT with a breath-hold technique enables a completely non-invasive approach to treat liver lesions while reducing the irradiated volume of the uninvolved healthy liver tissue. Furthermore, the ability of MR-linac systems to provide direct visualization of the patient anatomy throughout the treatment fraction can also reduce interfractional and intrafractional uncertainties in target localization and allow dose escalation strategies (85).

## Adaptive Approaches for Liver MRgRT

Adaptive radiotherapy (ART) emerged in the radiation therapy lexicon over 20 years ago, initially signifying a means to control daily set-up error using megavoltage portal imaging (86). However, the term now broadly signifies the process by which the delivered dose is monitored and modified during the course of treatment to ensure clinical acceptability and maximize clinical outcomes. Online ART specifically refers to the daily modification of the radiation treatment plan in response to observed changes in daily tumor and/or OARs anatomy, while the patient remains on the treatment table. This may adjust for tumor response (87) or inter-fraction tumor/OAR motion (51) and has the intent of maximizing the therapeutic index.

Online ART thus depends on high quality on-board imaging that is sufficient to visualize and delineate the target and/or OARs for daily plan re-optimization. Logically then, the clinical implementation of integrated MRgRT and MRI-guided online ART (MRgART) in 2014 (87) has led to the rapid expansion in use of online ART, including for liver tumors. MR-guided online adaptive radiotherapy can have several advantages over conventionally planned radiotherapy. The most established of these include target dose escalation, OAR dose reduction, and plan adjustment based on target response to treatment. Online adaptation through MRgART can allow target dose escalation and OAR dose reduction due to improved management of unpredictable inter-fraction motion. For patients with tumors near dynamic OARs, inter-fraction changes historically lead to uncertainty in the daily tumor/OAR geometric relationship. These uncertainties limit dose in order to maintain safety. In the upper abdomen, stomach filling, duodenal distension and motion, and small and large bowel motion may all lead to large changes in the proximity of liver targets to OARs (88). Online adaptation allows for daily plan adjustment in response to these changes to spare OAR dose while enabling confident delivery of ablative tumor dosing.

MRgART also enables plan changes to account for more predictable changes, such as tumor response over the course of therapy, or patient factors like weight loss or gain. However, it should be noted that given the additional time, personnel, and resources required to adapt a treatment plan at up to each fraction (89, 90), the tendency over the past six years of use has been implementation of MRgART mainly for SBRT or similarly hypo-fractionated courses, rather than for adaption for predictable changes occurring over a longer fractionation (91, 92). Thus, inter-fraction changes like day-to-day OAR motion are the more common driver of online adaptation in the current era, and most current data and experience with upper abdominal and liver MRgART is in this setting. As technology improves and time of delivery of MRgART shortens, use of MRgART may be more common in conventional fractionation schemas.

Given these specific advantages as well as the resources required for MRgART, patient selection is an important aspect of MRgART. With regard to the advantage of accounting for changing tumor/OAR geometry, tumors within 2 cm of the viscous gastrointestinal tract (*i.e.* peripheral liver tumors) are more likely to require plan adaptation than tumors surrounded by normal liver parenchyma (51, 52). This is due to the rapid dose fall-off with SBRT planning, wherein inter-fraction OAR motion has to occur within the higher dose gradients in order to meaningfully affect potential OAR dose. This may be particularly important in patients with liver metastases, where dose escalation has been linked with improved local control (93). While HCC may be successfully treated at somewhat lower SBRT doses, nearer to point dose tolerances of the stomach and bowel (94), large changes in stomach and OAR filling can be observed that exceed reasonable planning OAR volume (PRV) construction for avoidance (88). Therefore, peripheral HCCs may benefit from online adaptation to maintain adequate tumor dose while minimizing risk to OARs.

Other primary liver cancers, like cholangiocarcinomas, may also benefit from daily plan adaptation in order to mitigate potential OAR injury. This adaptation, which may in turn allow for safe tumor dose escalation, has been correlated with improved overall survival in cholangiocarcinoma patients (95). This style of dose escalation requires less intentional, conservative underdosing of the tumor rind adjacent to OARs and could be performed instead of the conventionally fractionated, multiple dose level, PRV approach that many centers use to attempt tumor dose escalation while protecting OARs. Similarly, in hilar cholangiocarcinomas, MRgART has been shown to minimize dose to the stomach and duodenum that can otherwise occur from daily changes in stomach and duodenal distension and positioning (52). This may allow further dose escalation in this challenging location, which may improve local control, a key element of either definitive or bridge-to-transplant therapy in these patients. Higher dose delivery may be feasible here, as the common bile duct (CBD) is often permanently stented in these patients, which may mitigate long term biliary stenosis (96), or can alternatively be in the setting of daily monitoring of dose to uninvolved duct, as the CBD is well-visualized on both 1.5 and 0.35 T on-board images (55).

MRgART also requires new considerations in workflow, which can be separated into: 1. Simulation, 2. On-table pre-treatment, and 3. Beam-on time-frames. At the time of this writing, there are two commercial integrated MRgRT platforms capable of online ART: the Elekta Unity 1.5 T device and the ViewRay MRIdian 0.35 T system. For simulation, computed tomography imaging is typically still obtained for density information for initial and subsequent adaptive plans. Patients can then be additionally imaged on the treatment MR-linac, which is helpful to learn how well target and OAR anatomy will be visualized ahead of the on-the-fly portion of adaptive re-planning. Standard immobilization can be used (as long as devices are MRI compatible), which can also help to minimize the need to online adapt simply to adjust for gross positional changes. Reproducibility of imaging coil positioning should also be considered, with options like building the coils into immobilization devices or custom table-overlays used variably between institutions.

In contrast to CBCT based IGRT, patients typically do not require fiducials, as the tumor is well-visualized on the available sequences of both devices. Intravenous contrast hepatobiliary contrast agents can be used for simulation and have also been shown to be safe for daily use in the setting of SBRT fractionation in patients with adequate renal function (84). Acquisition of simulation images both with and without contrast can help identify the cases in which it will be necessary for daily online ART fractions and, conversely, spare its daily use in those cases where it is less impactful.

The two commercial platforms share a similarly structured, on-table adaptive workflow, with minor differences (97). Of note, the 1.5 T system has two “adaptive” workflows, an “adapt to shape” workflow, which is akin to the definition of online adaptation used in this writing and used on the 0.35 T system, and an “adapt to position” workflow. The “adapt to position” workflow is essentially an isocenter shift (*via* a shift in MLCs) to overcome the inability to shift the patient couch on the 1.5 T system and is not the focus here.

On each platform, the on-table component of MRgART fractions is initiated by acquisition of the daily, online volumetric MRI. Typically, this sequence will match the sequence used at simulation to minimize impact of imaging differences on perceived changes in anatomy-of-the-day. Next, the pre-treatment planning image is rigidly registered to the image of the day, generally to the centroid of the gross liver tumor volume, or to adjacent surrogate structures if the tumor is difficult to visualize (vascular structures, liver edge, *etc*). On the 1.5 T system, this is achieved through export to a separate treatment planning system (TPS Monaco) (98). On the 0.35 T system, this is on the dedicated/integrated online MRIdian TPS (1).

Next, on both platforms, the original contours are automatically propagated to the daily image using rigid (preferred for targets, when possible) and/or deformable registration. Physicians and therapists then edit the contours as needed to match the daily anatomy. To save time in contouring, contour adjustments for SBRT plans may focus on anatomy within a 2–3cm ring around the PTV, which captures the high dose fall-off region of interest for OAR sparing and has been shown to be sufficient for robust and fast plan re-optimization (48). Electron density is updated, either through contour-based bulk density override (0.35 T system) or application of the average electron density for each structure as identified from the simulation image (1.5 T system). The plan is then reoptimized, typically through an adjustment of beam weighting, segments, and fluence or mix thereof, with maintenance of the original beam angles. In both planning systems, this process is rapid within the order of seconds to several minutes (51, 98).

Online, pre-treatment quality assurance is then performed (99), and the original plan is compared to the online adaptive plan, with selection of the superior plan for delivery. It is key to note that formal dosimetric (*e.g.* dose metric or DVH) comparison should be used for the selection of the superior plan, as visual assessment of the plan alone, for the need for daily adaptation has been shown to be inadequate for identifying fractions benefitting from plan change (100). The beam-on component of ART on both MRgART systems utilizes real-time cine MRI monitoring and beam-gating. On the 0.35 T system, cine MR imaging is at a rate of eight frames per second and beam-gating is automatic, through a deformable registration-based tracking algorithm (83). On the 1.5 T system, beam-gating is presently manual but still based on real time MRI target (or adjacent surrogate) monitoring (101). On both systems, breath-hold delivery may improve efficiency of treatment, and combinations of patient visual feedback and/or audio respiratory coaching have been utilized successfully. Specifics of motion management choices are discussed in more depth in the *Motion Management* section.

## Discussion

In the future, MRgART is likely to increase in both complexity and indication.

MR-only planning has been achieved in some settings (102) and may find ready application in liver patients in the setting of MRgRT. Future considerations also include personalized adaptation or dose prescribing based on MRI-specific imaging indications of tumor response, such as changes in diffusion restriction (103) or MRI tumor volumes during the course of treatment (104). However, standardization of imaging and methods for signal detection, as well as application to patient care, is needed for mainstream use (105). Ongoing and additional prospective clinical trial efforts are needed to establish the clinical benefit of MRgART in liver patients.

A future MRI-only liver SBRT workflow has advantages over the aforementioned CT–MR hybrid workflow, with the potential to improve overall efficiency. It requires replacement of planning CT with synthetic CT generated from the planning MRI (*i.e.* electron density mapping) through voxel-based methods, atlas based methods, or hybrid approach (106). This MRI-only workflow will reduce CT scanning to enable reduction of radiation dose and imaging costs with more efficient use of resources, and more importantly, avoid geometric uncertainties of MRI–CT co-registration through direct delineation of the target and OARs on MRI with improved geometric treatment accuracy (107). Inter- and intra-fractional treatment adaptation with fast auto-contouring algorithms, automated treatment planning, and automatic reconstruction of the delivered dose of the day cumulative dose delivered would facilitate and improve the accuracy of SBRT for liver cancer patients (108).

Several studies have demonstrated that higher doses of RT were correlated with improvement of tumor control and overall survival for many unresectable liver tumors (*i.e.* liver CRC metastases and hepatobiliary tumors) (40, 93, 95, 109). However the ability to deliver high-dose of RT to liver tumors adjacent to nearby luminal gastrointestinal organs and the requirement to spare sufficient un-involved liver to maintain synthetic liver function necessitate accurate liver cancer target delineation, precise RT planning, and real-time treatment adaptation to improve sustained local control while reducing the risk of toxicity. These challenges can be mitigated in part by MRI-based RT planning and delivery, when personalized dose escalation to liver tumors could be based on cumulative delivered doses to limiting OAR, rather than limiting the RT dose based on a single pre-treatment image.

MRgRT provides an elegant platform to investigate early biomarkers for tumor control and late toxicity, through repeated MR functional imaging obtained throughout a course of radiation therapy. DWI MRI is based upon differences in mobility of water protons in tissues and is useful for detection and characterization of focal liver lesion and assessment of tumor response to treatment. Advanced diffusion methods such as intravoxel incoherent motion (IVIM) may have potential for detection, staging, and evaluation of the progression of liver fibrosis and for liver lesion characterization (110). DWI has been studied as a potential imaging biomarker early during SBRT associated with long term local control. It has also been investigated as a biomarker for radiation related liver injury (103). Previous work has shown heterogeneous cell populations within individual tumors, and repeat DCE MRI scans throughout treatment were able to predict the change in hypoxia in preclinical model (111) Employing pre- and intra-treatment functional imaging provides an opportunity for further personalized treatment with optimization of SBRT dose on a daily basis to accommodate temporal heterogeneities in tumor, where SBRT dose escalation could target areas of highest biological resistance, while areas of good response undergo dose de-escalation, opening avenues for dose adaptation with improved therapeutic ratio.

Radiomics aims to utilize computational pipelines to extract the most informative features from radiological images routinely acquired in clinical settings. Recent computational advances have allowed deep neural networks (DNNs) to learn unique features with unprecedented performance for image classification (112), eliminating the need for hand-engineered features required for “conventional” radiomics analyses. The application of deep learning in the medical imaging field is in its infancy (112), with only a few studies that have applied DNN radiomics pipelines to predict patients’ clinical outcomes (113–119). The plethora of MR images generated through an MRgRT radiotherapy system would create very large datasets capable of similar, if not improved, utility given the more visualization provided by MRI. The use of MRI-derived data combined with correlative biologic factors (*e.g.* genomics, metabolomics) and tumor microenvironment information will provide more understanding of tumor biology, implicating heterogeneous tumor subpopulations and their surrounding microenvironment as key factors in clinical outcomes and allow for a substantial degree of treatment personalization.

## Author Contributions

Conceptualization: LB and LD. Writing original draft preparation: LB, SC, CG, LH, AH, and AR. Writing**—**review and editing: LB, SC, CG, LH, AH, and AR. Supervision: LD. All authors contributed to the article and approved the submitted version.

## Conflict of Interest

LB has active research agreements with ViewRay Inc and received speaker honoraria for scientific presentations. SC has received speaker fees and travel reimbursement from Elekta AB (Uppsala, Sweden). CG has received travel grants from Elekta AB (Stockholm, Sweden). The department of Radiation Oncology Tübingen received financial and technical support from Elekta AB (Stockholm, Sweden).

The remaining authors declare that the research was conducted in the absence of any commercial or financial relationships that could be construed as a potential conflict of interest.

## Abbreviations

ADC, Apparent diffusion coefficient; ART, Adaptive radiotherapy; ATP, Adapt-to-position; ATS, Adapt-to-shape; BCLC, Barcelona Clinic Liver Cancer; CBCT, Cone beam computed tomography; CBD, Common bile duct; CRC, Colorectal cancer; CT, Computed tomography; DCE, Dynamic contrast enhanced; DEB-TACE, Drug-eluting bead transarterial chemoembolization; DIBH, Deep inspiration breath hold; DNN, Deep neural networks; DP, Delayed phase; DVH, Dose-volume histogram; DWI, Diffusion weighted imaging; FFLP, Freedom from local progression; HBP, Hepatobiliary phase; HCC, Hepatocellular carcinoma; IBT, Interstitial brachytherapy; IGRT, Image guided radiation therapy; ITV, Internal target volume; IVIM, Intravoxel incoherent motion; kV, Kilo-voltage; LC, local control; LPFS, local progression free survival; MLL, metastatic liver lesions; MR, Magnetic resonance; MRgART, MRI-guided online adaptive radiotherapy; MRgRT, Magnetic resonance guided radiotherapy; MRI, Magnetic resonance imaging; MWA, Microwave ablation; NETs, Neuroendocrine tumors; OAR, Organ at risk; OS, Overall survival; PEI, Percutaneous ethanol injection; PRV, Planning organ at risk volume; PVP, Portal venous phase; PVTT, Portal vein tumor thrombosis; PTV, Planning target volume; RFA, Radiofrequency ablation; RILD, Radiation-induced liver disease; RT, Radiation therapy; SBRT, Stereotactic body radiation therapy; SMART, Stereotactic MR-guided adaptive radiation therapy; T1WI, T1 weighted image; T2WI, T2 weighted image; TACE, Transarterial chemoembolization; TPS, Treatment planning system; TRUFI, True fast imaging.

## References

[1] Mutic S Dempsey JF. The ViewRay system: magnetic resonance-guided and controlled radiotherapy. Semin Radiat Oncol (2014) 24:196–9. 10.1016/j.semradonc.2014.02.008 24931092

[2] Klüter S. Technical design and concept of a 0.35 T MR-Linac. Clin Trans Radiat Oncol (2019) 18:98–101. 10.1016/j.ctro.2019.04.007 PMC6630153 31341983

[3] Raaymakers BW Lagendijk JJW Overweg J Kok JGM Raaijmakers AJE Kerkhof EM. Integrating a 1.5 T MRI scanner with a 6 MV accelerator: proof of concept. Phys Med Biol (2009) 54:N229–237. 10.1088/0031-9155/54/12/N01 19451689

[4] Corradini S Alongi F Andratschke N Belka C Boldrini L Cellini F. MR-guidance in clinical reality: current treatment challenges and future perspectives. Radiat Oncol (2019) 14:92. 10.1186/s13014-019-1308-y 31167658 PMC6551911

[5] Rudra S Jiang N Rosenberg SA Olsen JR Roach MC Wan L. Using adaptive magnetic resonance image-guided radiation therapy for treatment of inoperable pancreatic cancer. Cancer Med (2019) 8:2123–32. 10.1002/cam4.2100 PMC6536981 30932367

[6] Tetar SU Bruynzeel AME Oei SS Senan S Fraikin T Slotman BJ. Magnetic Resonance-guided Stereotactic Radiotherapy for Localized Prostate Cancer: Final Results on Patient-reported Outcomes of a Prospective Phase 2 Study. Eur Urol Oncol (2020) S2588-9311(20)30061-4. 10.1016/j.euo.2020.05.007 32536573

[7] Hall WA Paulson ES van der Heide UA Fuller CD Raaymakers BW Lagendijk JJW. The transformation of radiation oncology using real-time magnetic resonance guidance: A review. Eur J Cancer (2019) 122:42–52. 10.1016/j.ejca.2019.07.021 31614288 PMC8447225

[8] Witt JS Rosenberg SA Bassetti MF. MRI-guided adaptive radiotherapy for liver tumours: visualising the future. Lancet Oncol (2020) 21:e74–82. 10.1016/S1470-2045(20)30034-6 32007208

[9] Choi SH Seong J. Strategic application of radiotherapy for hepatocellular carcinoma. Clin Mol Hepatol (2018) 24:114–34. 10.3350/cmh.2017.0073 PMC6038936 29439305

[10] Mahadevan A Blanck O Lanciano R Peddada A Sundararaman S D’Ambrosio D. Stereotactic Body Radiotherapy (SBRT) for liver metastasis – clinical outcomes from the international multi-institutional RSSearch® Patient Registry. Radiat Oncol (2018) 13(1):26. 10.1186/s13014-018-0969-2 29439707 PMC5811977

[11] Rosenberg SA Henke LE Shaverdian N Mittauer K Wojcieszynski AP Hullett CR. A Multi-Institutional Experience of MR-Guided Liver Stereotactic Body Radiation Therapy. Adv Radiat Oncol (2019) 4:142–9. 10.1016/j.adro.2018.08.005 PMC6349638 30706022

[12] Nair VJ Pantarotto JR. Treatment of metastatic liver tumors using stereotactic ablative radiotherapy. World J Radiol (2014) 6:18–25. 10.4329/wjr.v6.i2.18 24578789 PMC3935063

[13] Yang DS Yoon WS Lee JA Lee NK Lee S Kim CY. The effectiveness of gadolinium MRI to improve target delineation for radiotherapy in hepatocellular carcinoma: a comparative study of rigid image registration techniques. Phys Med (2014) 30:676–81. 10.1016/j.ejmp.2014.05.003 24870246

[14] Guckenberger M Lievens Y Bouma AB Collette L Dekker A deSouza NM. Characterisation and classification of oligometastatic disease: a European Society for Radiotherapy and Oncology and European Organisation for Research and Treatment of Cancer consensus recommendation. Lancet Oncol (2020) 21:e18–28. 10.1016/S1470-2045(19)30718-1 31908301

[15] Mazzaferro V Regalia E Doci R Andreola S Pulvirenti A Bozzetti F. Liver transplantation for the treatment of small hepatocellular carcinomas in patients with cirrhosis. N Engl J Med (1996) 334:693–9. 10.1056/NEJM199603143341104 8594428

[16] Lin C-W Chen Y-S Lin C-C Lee P-H Lo G-H Hsu C-C. Significant predictors of overall survival in patients with hepatocellular carcinoma after surgical resection. PloS One (2018) 13:e0202650. 10.1371/journal.pone.0202650 30180193 PMC6122804

[17] Cabibbo G Enea M Attanasio M Bruix J Craxì A Cammà C. A meta-analysis of survival rates of untreated patients in randomized clinical trials of hepatocellular carcinoma. Hepatology (2010) 51:1274–83. 10.1002/hep.23485 20112254

[18] Yang Y Si T. Yttrium-90 transarterial radioembolization versus conventional transarterial chemoembolization for patients with hepatocellular carcinoma: a systematic review and meta-analysis. Cancer Biol Med (2018) 15:299–310. 10.20892/j.issn.2095-3941.2017.0177 30197797 PMC6121048

[19] Zhang Y Hu J Li J Wang N Li W Zhou Y. Comparison of imaging-based gross tumor volume and pathological volume determined by whole-mount serial sections in primary cervical cancer. Onco Targets Ther (2013) 6:917–23. 10.2147/OTT.S43264 PMC3722137 23888117

[20] Dawson LA Normolle D Balter JM McGinn CJ Lawrence TS Ten Haken RK. Analysis of radiation-induced liver disease using the Lyman NTCP model. Int J Radiat Oncol Biol Phys (2002) 53:810–21. 10.1016/S0360-3016(02)02846-8 12095546

[21] European Association for the Study of the Liver Electronic address: easloffice@easloffice.eu, European Association for the Study of the Liver. EASL Clinical Practice Guidelines: Management of hepatocellular carcinoma. J Hepatol (2018) 69:182–236. 10.1016/j.jhep.2018.03.019 29628281

[22] Kopek N Holt MI Hansen AT Høyer M. Stereotactic body radiotherapy for unresectable cholangiocarcinoma. Radiother Oncol (2010) 94:47–52. 10.1016/j.radonc.2009.11.004 19963295

[23] Zeng Z-C Seong J Yoon SM Cheng JC-H Lam K-O Lee A-S. Consensus on Stereotactic Body Radiation Therapy for Small-Sized Hepatocellular Carcinoma at the 7th Asia-Pacific Primary Liver Cancer Expert Meeting. Liver Cancer (2017) 6:264–74. 10.1159/000475768 PMC5704685 29234630

[24] Hara K Takeda A Tsurugai Y Saigusa Y Sanuki N Eriguchi T. Radiotherapy for Hepatocellular Carcinoma Results in Comparable Survival to Radiofrequency Ablation: A Propensity Score Analysis. Hepatology (2019) 69:2533–45. 10.1002/hep.30591 30805950

[25] Huo YR Eslick GD. Transcatheter Arterial Chemoembolization Plus Radiotherapy Compared With Chemoembolization Alone for Hepatocellular Carcinoma: A Systematic Review and Meta-analysis. JAMA Oncol (2015) 1:756–65. 10.1001/jamaoncol.2015.2189 26182200

[26] Moon DH Wang AZ Tepper JE. A prospective study of the safety and efficacy of liver stereotactic body radiotherapy in patients with and without prior liver-directed therapy. Radiother Oncol (2018) 126:527–33. 10.1016/j.radonc.2018.01.004 29366521

[27] Murray LJ Dawson LA. Advances in Stereotactic Body Radiation Therapy for Hepatocellular Carcinoma. Semin Radiat Oncol (2017) 27:247–55. 10.1016/j.semradonc.2017.02.002 28577832

[28] Lasley FD Mannina EM Johnson CS Perkins SM Althouse S Maluccio M. Treatment variables related to liver toxicity in patients with hepatocellular carcinoma, Child-Pugh class A and B enrolled in a phase 1-2 trial of stereotactic body radiation therapy. Pract Radiat Oncol (2015) 5:e443–9. 10.1016/j.prro.2015.02.007 25899219

[29] Andolino DL Johnson CS Maluccio M Kwo P Tector AJ Zook J. Stereotactic Body Radiotherapy for Primary Hepatocellular Carcinoma. Int J Radiat Oncol Biol Phys (2011) 81:e447–53. 10.1016/j.ijrobp.2011.04.011 21645977

[30] Kong X Dong Y Wu J He J Le Y Du K. High-biologically effective dose palliative radiotherapy for a tumor thrombus might improve the long-term prognosis of hepatocellular carcinoma: a retrospective study. Radiat Oncol (2017) 12:92. 10.1186/s13014-017-0831-y 28569169 PMC5452386

[31] Xi M Zhang L Zhao L Li Q-Q Guo S-P Feng Z-Z. Effectiveness of Stereotactic Body Radiotherapy for Hepatocellular Carcinoma with Portal Vein and/or Inferior Vena Cava Tumor Thrombosis. PloS One (2013) 8:e63864. 10.1371/journal.pone.0063864 23737955 PMC3667854

[32] Chen S-W Lin L-C Kuo Y-C Liang J-A Kuo C-C Chiou J-F. Phase 2 Study of Combined Sorafenib and Radiation Therapy in Patients With Advanced Hepatocellular Carcinoma. Int J Radiat Oncol Biol Phys (2014) 88:1041–7. 10.1016/j.ijrobp.2014.01.017 24661657

[33] Pollom EL Deng L Pai RK Brown JM Giaccia A Loo BW. Gastrointestinal Toxicities With Combined Antiangiogenic and Stereotactic Body Radiation Therapy. Int J Radiat Oncol Biol Phys (2015) 92:568–76. 10.1016/j.ijrobp.2015.02.016 PMC4816453 26068491

[34] Brade AM Ng S Brierley J Kim J Dinniwell R Ringash J. Phase 1 Trial of Sorafenib and Stereotactic Body Radiation Therapy for Hepatocellular Carcinoma. Int J Radiat Oncol Biol Phys (2016) 94:580–7. 10.1016/j.ijrobp.2015.11.048 26867886

[35] Finn RS Qin S Ikeda M Galle PR Ducreux M Kim T-Y. Atezolizumab plus Bevacizumab in Unresectable Hepatocellular Carcinoma. N Engl J Med (2020) 382:1894–905. 10.1056/NEJMoa1915745 32402160

[36] Frakulli R Buwenge M Macchia G Cammelli S Deodato F Cilla S. Stereotactic body radiation therapy in cholangiocarcinoma: a systematic review. Br J Radiol (2019) 92:20180688. 10.1259/bjr.20180688 30673295 PMC6580923

[37] Herfarth KK Debus J Lohr F Bahner ML Rhein B Fritz P. Stereotactic Single-Dose Radiation Therapy of Liver Tumors: Results of a Phase I/II Trial. J Clin Oncol (2016) 19(1):164–70. 10.1200/JCO.2001.19.1.164 11134209

[38] Méndez Romero A Wunderink W Hussain SM De Pooter JA Heijmen BJM Nowak PCJM. Stereotactic body radiation therapy for primary and metastatic liver tumors: A single institution phase i-ii study. Acta Oncol (2006) 45:831–7. 10.1080/02841860600897934 16982547

[39] Hoyer M Roed H Traberg Hansen A Ohlhuis L Petersen J Nellemann H. Phase II study on stereotactic body radiotherapy of colorectal metastases. Acta Oncol (2006) 45:823–30. 10.1080/02841860600904854 16982546

[40] Rusthoven KE Kavanagh BD Cardenes H Stieber VW Burri SH Feigenberg SJ. Multi-institutional phase I/II trial of stereotactic body radiation therapy for liver metastases. J Clin Oncol (2009) 27:1572–8. 10.1200/JCO.2008.19.6329 19255321

[41] Scorsetti M Arcangeli S Tozzi A Comito T Alongi F Navarria P. Is stereotactic body radiation therapy an attractive option for unresectable liver metastases? A preliminary report from a phase 2 trial. Int J Radiat Oncol Biol Phys (2013) 86:336–42. 10.1016/j.ijrobp.2012.12.021 23433800

[42] Comito T Clerici E Tozzi A D’Agostino G. Liver metastases and SBRT: A new paradigm? Rep Pract Oncol Radiother (2015) 20:464–71. 10.1016/j.rpor.2014.10.002 PMC4661346 26696787

[43] Franzese C Comito T Clerici E Di Brina L Tomatis S Navarria P. Liver metastases from colorectal cancer: propensity score-based comparison of stereotactic body radiation therapy vs. microwave ablation. J Cancer Res Clin Oncol (2018) 144:1777–83. 10.1007/s00432-018-2692-7 PMC11813392 29934790

[44] Jackson WC Tao Y Mendiratta-Lala M Bazzi L Wahl DR Schipper MJ. Comparison of Stereotactic Body Radiation Therapy and Radiofrequency Ablation in the Treatment of Intrahepatic Metastases. Int J Radiat Oncol Biol Phys (2018) 100:950–8. 10.1016/j.ijrobp.2017.12.014 PMC6142177 29485074

[45] Barry A Wong R Dawson LA. The Management of Colorectal Cancer Liver Metastases: The Radiation Oncology Viewpoint. Int J Radiat Oncol Biol Phys (2019) 103:540–1. 10.1016/j.ijrobp.2018.10.010 30722966

[46] Feldman AM Modh A Glide-Hurst C Chetty IJ Movsas B. Real-time Magnetic Resonance-guided Liver Stereotactic Body Radiation Therapy: An Institutional Report Using a Magnetic Resonance-Linac System. Cureus (2019) 11:e5774. 10.7759/cureus.5774 31723533 PMC6825488

[47] Henke LE Olsen JR Contreras JA Curcuru A DeWees TA Green OL. Stereotactic MR-Guided Online Adaptive Radiation Therapy (SMART) for Ultracentral Thorax Malignancies: Results of a Phase 1 Trial. Adv Radiat Oncol (2019) 4:201–9. 10.1016/j.adro.2018.10.003 PMC6349650 30706029

[48] Bohoudi O Bruynzeel AME Senan S Cuijpers JP Slotman BJ Lagerwaard FJ. Fast and robust online adaptive planning in stereotactic MR-guided adaptive radiation therapy (SMART) for pancreatic cancer. Radiother Oncol (2017) 125:439–44. 10.1016/j.radonc.2017.07.028 28811038

[49] Hal WA Straza MW Chen X Mickevicius N Erickson B Schultz C. Initial clinical experience of Stereotactic Body Radiation Therapy (SBRT) for liver metastases, primary liver malignancy, and pancreatic cancer with 4D-MRI based online adaptation and real-time MRI monitoring using a 1.5 Tesla MR-Linac. PloS One (2020) 15(8). 10.1371/journal.pone.0236570 PMC7413561 32764748

[50] Klüter S Katayama S Spindeldreier CK Koerber SA Major G Alber M. First prospective clinical evaluation of feasibility and patient acceptance of magnetic resonance-guided radiotherapy in Germany. Strahlenther Onkol (2020) 196:691–8. 10.1007/s00066-020-01578-z PMC7385000 32002567

[51] Henke L Kashani R Robinson C Curcuru A DeWees T Bradley J. Phase I trial of stereotactic MR-guided online adaptive radiation therapy (SMART) for the treatment of oligometastatic or unresectable primary malignancies of the abdomen. Radiother Oncol (2018) 126:519–26. 10.1016/j.radonc.2017.11.032 29277446

[52] Luterstein E Cao M Lamb JM Raldow A Low D Steinberg ML. Clinical Outcomes Using Magnetic Resonance–Guided Stereotactic Body Radiation Therapy in Patients With Locally Advanced Cholangiocarcinoma. Adv Radiat Oncol (2020) 5:189–95. 10.1016/j.adro.2019.09.008 PMC7136637 32280818

[53] Boldrini L Romano A Mariani S Cusumano D Catucci F Placidi L. MRI-guided stereotactic radiation therapy for hepatocellular carcinoma: a feasible and safe innovative treatment approach. J Cancer Res Clin Oncol (2021). 10.1007/s00432-020-03480-8 PMC11801863 33398447

[54] Centre Georges Francois Leclerc. Phase II of Adaptative Magnetic Resonance-Guided Stereotactic Body Radiotherapy (SBRT) for Treatment of Primary or Secondary Progressive Liver Tumors. clinicaltrials.gov (2020). Available at: https://clinicaltrials.gov/ct2/show/NCT04242342 (Accessed March 10, 2021).

[55] Lukovic J Henke L Gani C Kim TK Stanescu T Hosni A. MRI-Based Upper Abdominal Organs-at-Risk Atlas for Radiation Oncology. Int J Radiat Oncol Biol Phys (2020) 106:743–53. 10.1016/j.ijrobp.2019.12.003 31953061

[56] Goodwin MD Dobson JE Sirlin CB Lim BG Stella DL. Diagnostic challenges and pitfalls in MR imaging with hepatocyte-specific contrast agents. Radiographics (2011) 31:1547–68. 10.1148/rg.316115528 21997981

[57] Nair VJ Szanto J Vandervoort E Henderson E Avruch L Malone S. Feasibility, detectability and clinical experience with platinum fiducial seeds for MRI/CT fusion and real-time tumor tracking during CyberKnife® stereotactic ablative radiotherapy†. J Radiosurg SBRT (2015) 3:315–23. PMC5675499 29296414

[58] Lencioni R Cioni D Della Pina C Crocetti L Bartolozzi C. Imaging diagnosis. Semin Liver Dis (2005) 25:162–70. 10.1055/s-2005-871196 15918145

[59] Marrero JA Hussain HK Nghiem HV Umar R Fontana RJ Lok AS. Improving the prediction of hepatocellular carcinoma in cirrhotic patients with an arterially-enhancing liver mass. Liver Transpl (2005) 11:281–9. 10.1002/lt.20357 15719410

[60] Matos AP Velloni F Ramalho M AlObaidy M Rajapaksha A Semelka RC. Focal liver lesions: Practical magnetic resonance imaging approach. World J Hepatol (2015) 7:1987–2008. 10.4254/wjh.v7.i16.1987 26261689 PMC4528273

[61] Zech CJ Herrmann KA Reiser MF Schoenberg SO. MR imaging in patients with suspected liver metastases: value of liver-specific contrast agent Gd-EOB-DTPA. Magn Reson Med Sci (2007) 6:43–52. 10.2463/mrms.6.43 17510541

[62] Danet I-M Semelka RC Leonardou P Braga L Vaidean G Woosley JT. Spectrum of MRI appearances of untreated metastases of the liver. AJR Am J Roentgenol (2003) 181:809–17. 10.2214/ajr.181.3.1810809 12933487

[63] Namasivayam S Martin DR Saini S. Imaging of liver metastases: MRI. Cancer Imaging (2007) 7:2–9. 10.1102/1470-7330.2007.0002 17293303 PMC1804118

[64] Sica GT Ji H Ros PR. Computed tomography and magnetic resonance imaging of hepatic metastases. Clin Liver Dis (2002) 6:165–179, vii. 10.1016/s1089-3261(03)00071-0 11933587

[65] Kanematsu M Goshima S Watanabe H Kondo H Kawada H Noda Y. Diffusion/perfusion MR imaging of the liver: practice, challenges, and future. Magn Reson Med Sci (2012) 11:151–61. 10.2463/mrms.11.151 23037559

[66] Vilgrain V Esvan M Ronot M Caumont-Prim A Aubé C Chatellier G. A meta-analysis of diffusion-weighted and gadoxetic acid-enhanced MR imaging for the detection of liver metastases. Eur Radiol (2016) 26:4595–615. 10.1007/s00330-016-4250-5 26883327

[67] Toesca DAS Osmundson EC von Eyben R Shaffer JL Lu P Koong AC. Central liver toxicity after SBRT: An expanded analysis and predictive nomogram. Radiother Oncol (2017) 122:130–6. 10.1016/j.radonc.2016.10.024 27865544

[68] Powerski M Penzlin S Hass P Seidensticker R Mohnike K Damm R. Biliary duct stenosis after image-guided high-dose-rate interstitial brachytherapy of central and hilar liver tumors: A systematic analysis of 102 cases. Strahlenther Und Onkol (2019) 195:265–73. 10.1007/s00066-018-1404-1 30470846

[69] Abbas H Chang B Chen Z. Motion management in gastrointestinal cancers. J Gastrointest Oncol (2014) 5:223–35. 10.3978/j.issn.2078-6891.2014.028 PMC4074952 24982771

[70] Langen KM Jones DT. Organ motion and its management. Int J Radiat Oncol Biol Phys (2001) 50:265–78. 10.1016/s0360-3016(01)01453-5 11316572

[71] Gargett M Haddad C Kneebone A Booth JT Hardcastle N. Clinical impact of removing respiratory motion during liver SABR. Radiat Oncol (2019) 14:1–9. 10.1186/s13014-019-1300-6 31159840 PMC6547575

[72] Ricke J Klümpen HJ Amthauer H Bargellini I Bartenstein P de Toni EN. Impact of combined selective internal radiation therapy and sorafenib on survival in advanced hepatocellular carcinoma. J Hepatol (2019) 71:1164–74. 10.1016/j.jhep.2019.08.006 31421157

[73] Corradini S Alongi F Andratschke N Belka C Boldrini L Cellini F. MR-guidance in clinical reality: current treatment challenges and future perspectives. Radiat Oncol (2019) 1492. 10.1186/s13014-019-1308-y PMC6551911 31167658

[74] Choi GW Suh Y Das P Herman J Holliday E Koay E. Assessment of setup uncertainty in hypofractionated liver radiation therapy with a breath-hold technique using automatic image registration-based image guidance. Radiat Oncol (2019) 14:1–9. 10.1186/s13014-019-1361-6 31470860 PMC6717376

[75] Heinz C Gerum S Freislederer P Ute G Roeder F Stefanie Corradini CB. Feasibility study on image guided patient positioning for stereotactic body radiation therapy of liver malignancies guided by liver motion. Radiat Oncol (2016) 11:1–7. 10.1186/s13014-016-0662-2 27350636 PMC4924279

[76] van de Lindt TN Fast MF van Kranen SR Nowee ME Jansen EPM van der Heide UA. MRI-guided mid-position liver radiotherapy: Validation of image processing and registration steps. Radiother Oncol (2019) 138:132–40. 10.1016/j.radonc.2019.06.007 31252295

[77] Kurz C Buizza G Landry G Kamp F Rabe M Paganelli C. Medical physics challenges in clinical MR-guided radiotherapy. Radiat Oncol (2020) 15:1–16. 10.1186/s13014-020-01524-4 PMC7201982 32370788

[78] Jackson S Glitzner M Tijssen RHN Raaymakers BW. MRI B (0) homogeneity and geometric distortion with continuous linac gantry rotation on an Elekta Unity MR-linac. Phys Med Biol (2019) 64:12NT01. 10.1088/1361-6560/ab231a 31108467

[79] Boda-Heggemann J Knopf AC Simeonova-Chergou A Wertz H Stieler F Jahnke A. Deep Inspiration Breath Hold-Based Radiation Therapy: A Clinical Review. Int J Radiat Oncol Biol Phys (2016) 94:478–92. 10.1016/j.ijrobp.2015.11.049 26867877

[80] Eccles C Brock KK Bissonnette J-P Hawkins M Dawson LA. Reproducibility of liver position using active breathing coordinator for liver cancer radiotherapy. Int J Radiat Oncol Biol Phys (2006) 64:751–9. 10.1016/j.ijrobp.2005.05.066 16458774

[81] Kim J Lee H Wu H-G Chie EK Kang H-C Park JM. Development of patient-controlled respiratory gating system based on visual guidance for magnetic-resonance image-guided radiation therapy. Med Phys (2017) 44:4838–46. 10.1002/mp.12447 28675492

[82] van Sörnsen de Koste JR Palacios MA Bruynzeel AME Slotman BJ Senan S Lagerwaard FJ. MR-guided Gated Stereotactic Radiation Therapy Delivery for Lung, Adrenal, and Pancreatic Tumors: A Geometric Analysis. Int J Radiat Oncol Biol Phys (2018) 102:858–66. 10.1016/j.ijrobp.2018.05.048 30061007

[83] Green OL Rankine LJ Cai B Curcuru A Kashani R Rodriguez V. First clinical implementation of real-time, real anatomy tracking and radiation beam control. Med Phys (2018) 45:3728–40. 10.1002/mp.13002 29807390

[84] Wojcieszynski AP Rosenberg SA Brower JV Hullett CR Geurts MW Labby ZE. Gadoxetate for direct tumor therapy and tracking with real-time MRI-guided stereotactic body radiation therapy of the liver. Radiother Oncol (2016) 118:416–8. 10.1016/j.radonc.2015.10.024 26627702

[85] Bruynzeel AME Lagerwaard FJ. The role of biological dose-escalation for pancreatic cancer. Clin Trans Radiat Oncol (2019) 18:128–30. 10.1016/j.ctro.2019.04.020 PMC6630149 31341988

[86] Yan D Vicini F Wong J Martinez A. Adaptive radiation therapy. Phys Med Biol (1997) 42:123–32. 10.1088/0031-9155/42/1/008 9015813

[87] Acharya S Fischer-Valuck BW Mazur TR Curcuru A Sona K Kashani R. Magnetic Resonance Image Guided Radiation Therapy for External Beam Accelerated Partial-Breast Irradiation: Evaluation of Delivered Dose and Intrafractional Cavity Motion. Int J Radiat Oncol Biol Phys (2016) 96:785–92. 10.1016/j.ijrobp.2016.08.006 27788951

[88] Henke L Kashani R Yang D Zhao T Green O Olsen L. Simulated Online Adaptive Magnetic Resonance-Guided Stereotactic Body Radiation Therapy for the Treatment of Oligometastatic Disease of the Abdomen and Central Thorax: Characterization of Potential Advantages. Int J Radiat Oncol Biol Phys (2016) 96:1078–86. 10.1016/j.ijrobp.2016.08.036 PMC5376349 27742541

[89] Mittauer K Paliwal B Hill P Bayouth JE Geurts MW Baschnagel AM. A New Era of Image Guidance with Magnetic Resonance-guided Radiation Therapy for Abdominal and Thoracic Malignancies. Cureus (2018) 10:e2422. 10.7759/cureus.2422 29872602 PMC5985918

[90] Tetar SU Bruynzeel AME Lagerwaard FJ Slotman BJ Bohoudi O Palacios MA. Clinical implementation of magnetic resonance imaging guided adaptive radiotherapy for localized prostate cancer. Phys Imaging Radiat Oncol (2019) 8:9:69–76. 10.1016/j.phro.2019.02.002 PMC7807673 33458428

[91] Henke LE Contreras JA Green OL Cai B Kim H Roach MC. Magnetic Resonance Image-Guided Radiotherapy (MRIgRT): A 4.5-Year Clinical Experience. Clin Oncol (R Coll Radiol) (2018) 30:720–7. 10.1016/j.clon.2018.08.010 PMC6177300 30197095

[92] Werensteijn-Honingh AM Kroon PS Winkel D Aalbers EM van Asselen B Bol GH. Feasibility of stereotactic radiotherapy using a 1.5T MR-linac: Multi-fraction treatment of pelvic lymph node oligometastases. Radiother Oncol (2019) 134:50–4. 10.1016/j.radonc.2019.01.024 31005224

[93] Rule W Timmerman R Tong L Abdulrahman R Meyer J Boike T. Phase I dose-escalation study of stereotactic body radiotherapy in patients with hepatic metastases. Ann Surg Oncol (2011) 18:1081–7. 10.1245/s10434-010-1405-5 21046264

[94] Sanuki N Takeda A Oku Y Mizuno T Aoki Y Eriguchi T. Stereotactic body radiotherapy for small hepatocellular carcinoma: A retrospective outcome analysis in 185 patients. Acta Oncol (2014) 53:399–404. 10.3109/0284186X.2013.820342 23962244

[95] Tao R Krishnan S Bhosale PR Javle MM Aloia TA Shroff RT. Ablative Radiotherapy Doses Lead to a Substantial Prolongation of Survival in Patients With Inoperable Intrahepatic Cholangiocarcinoma: A Retrospective Dose Response Analysis. J Clin Oncol (2016) 34:219–26. 10.1200/JCO.2015.61.3778 PMC4980564 26503201

[96] Sandler KA Veruttipong D Agopian VG Finn RS Hong JC Kaldas FM. Stereotactic body radiotherapy (SBRT) for locally advanced extrahepatic and intrahepatic cholangiocarcinoma. Adv Radiat Oncol (2016) 1:237–43. 10.1016/j.adro.2016.10.008 PMC5514222 28740893

[97] Green OL Henke LE Hugo GD. Practical Clinical Workflows for Online and Offline Adaptive Radiation Therapy. Semin Radiat Oncol (2019) 29:219–27. 10.1016/j.semradonc.2019.02.004 PMC6487881 31027639

[98] Winkel D Bol GH Kroon PS van Asselen B Hackett SS Werensteijn-Honingh AM. Adaptive radiotherapy: The Elekta Unity MR-linac concept. Clin Trans Radiat Oncol (2019) 18:54–9. 10.1016/j.ctro.2019.04.001 PMC6630157 31341976

[99] Chen X Ahunbay E Paulson ES Chen G Li XA. A daily end-to-end quality assurance workflow for MR-guided online adaptive radiation therapy on MR-Linac. J Appl Clin Med Phys (2020) 21:205–12. 10.1002/acm2.12786 PMC6964761 31799753

[100] Tyran M Jiang N Cao M Raldow A Lamb JM Low D. Retrospective evaluation of decision-making for pancreatic stereotactic MR-guided adaptive radiotherapy. Radiother Oncol (2018) 129:319–25. 10.1016/j.radonc.2018.08.009 30174107

[101] Keiper TD Tai A Chen X Paulson E Lathuilière F Bériault S. Feasibility of real-time motion tracking using cine MRI during MR-guided radiation therapy for abdominal targets. Med Phys (2020) 47:3554–66. 10.1002/mp.14230 32402111

[102] Tenhunen M Korhonen J Kapanen M Seppälä T Koivula L Collan J. MRI-only based radiation therapy of prostate cancer: workflow and early clinical experience. Acta Oncol (2018) 57:902–7. 10.1080/0284186X.2018.1445284 29488426

[103] Eccles CL Haider EA Haider MA Fung S Lockwood G Dawson LA. Change in diffusion weighted MRI during liver cancer radiotherapy: preliminary observations. Acta Oncol (2009) 48:1034–43. 10.1080/02841860903099972 19634060

[104] Cusumano D Boldrini L Yadav P Yu G Musurunu B Chiloiro G. External Validation of Early Regression Index (ERITCP) as Predictor of Pathologic Complete Response in Rectal Cancer Using Magnetic Resonance-Guided Radiation Therapy. Int J Radiat Oncol Biol Phys (2020) 108(5):1347–56. 10.1016/j.ijrobp.2020.07.2323 32758641

[105] Shukla-Dave A Obuchowski NA Chenevert TL Jambawalikar S Schwartz LH Malyarenko D. Quantitative imaging biomarkers alliance (QIBA) recommendations for improved precision of DWI and DCE-MRI derived biomarkers in multicenter oncology trials. J Magn Reson Imaging (2019) 49:e101–21. 10.1002/jmri.26518 PMC6526078 30451345

[106] Johnstone E Wyatt JJ Henry AM Short SC Sebag-Montefiore D Murray L. Systematic Review of Synthetic Computed Tomography Generation Methodologies for Use in Magnetic Resonance Imaging-Only Radiation Therapy. Int J Radiat Oncol Biol Phys (2018) 100:199–217. 10.1016/j.ijrobp.2017.08.043 29254773

[107] Edmund JM Nyholm T. A review of substitute CT generation for MRI-only radiation therapy. Radiat Oncol (2017) 12:28. 10.1186/s13014-016-0747-y 28126030 PMC5270229

[108] Menten MJ Mohajer JK Nilawar R Bertholet J Dunlop A Pathmanathan AU. Automatic reconstruction of the delivered dose of the day using MR-linac treatment log files and online MR imaging. Radiother Oncol (2020) 145:88–94. 10.1016/j.radonc.2019.12.010 31931291 PMC7191265

[109] Ben-Josef E Normolle D Ensminger WD Walker S Tatro D Ten Haken RK. Phase II trial of high-dose conformal radiation therapy with concurrent hepatic artery floxuridine for unresectable intrahepatic malignancies. J Clin Oncol (2005) 23:8739–47. 10.1200/JCO.2005.01.5354 16314634

[110] Lewis S Dyvorne H Cui Y Taouli B. Diffusion-Weighted Imaging of the Liver: Techniques and Applications. Magn Reson Imaging Clin N Am (2014) 22:373–95. 10.1016/j.mric.2014.04.009 PMC4121599 25086935

[111] Taylor E Zhou J Lindsay P Foltz W Cheung M Siddiqui I. Quantifying Reoxygenation in Pancreatic Cancer During Stereotactic Body Radiotherapy. Sci Rep (2020) 10:1638. 10.1038/s41598-019-57364-0 32005829 PMC6994660

[112] Litjens G Kooi T Bejnordi BE Setio AAA Ciompi F Ghafoorian M. A survey on deep learning in medical image analysis. Med Image Anal (2017) 42:60–88. 10.1016/j.media.2017.07.005 28778026

[113] Oakden-Rayner L Carneiro G Bessen T Nascimento JC Bradley AP Palmer LJ. Precision Radiology: Predicting longevity using feature engineering and deep learning methods in a radiomics framework. Sci Rep (2017) 7:1648. 10.1038/s41598-017-01931-w 28490744 PMC5431941

[114] Bibault J-E Giraud P Housset M Durdux C Taieb J Berger A. Deep Learning and Radiomics predict complete response after neo-adjuvant chemoradiation for locally advanced rectal cancer. Sci Rep (2018) 8:12611. 10.1038/s41598-018-30657-6 30135549 PMC6105676

[115] Wang K Lu X Zhou H Gao Y Zheng J Tong M. Deep learning Radiomics of shear wave elastography significantly improved diagnostic performance for assessing liver fibrosis in chronic hepatitis B: a prospective multicentre study. Gut (2019) 68:729–41. 10.1136/gutjnl-2018-316204 PMC6580779 29730602

[116] Choi JY. Radiomics and Deep Learning in Clinical Imaging: What Should We Do? Nucl Med Mol Imaging (2018) 52:89–90. 10.1007/s13139-018-0514-0 29662556 PMC5897263

[117] Lao J Chen Y Li Z-C Li Q Zhang J Liu J. A Deep Learning-Based Radiomics Model for Prediction of Survival in Glioblastoma Multiforme. Sci Rep (2017) 7:10353. 10.1038/s41598-017-10649-8 28871110 PMC5583361

[118] Li Z Wang Y Yu J Guo Y Cao W. Deep Learning based Radiomics (DLR) and its usage in noninvasive IDH1 prediction for low grade glioma. Sci Rep (2017) 7:5467. 10.1038/s41598-017-05848-2 28710497 PMC5511238

[119] Kickingereder P Isensee F Tursunova I Petersen J Neuberger U Bonekamp D. Automated quantitative tumour response assessment of MRI in neuro-oncology with artificial neural networks: a multicentre, retrospective study. Lancet Oncol (2019) 20:728–40. 10.1016/S1470-2045(19)30098-1 30952559

